# Predation on the Invasive Copepod, *Pseudodiaptomus forbesi*, and Native Zooplankton in the Lower Columbia River: An Experimental Approach to Quantify Differences in Prey-Specific Feeding Rates

**DOI:** 10.1371/journal.pone.0144095

**Published:** 2015-11-30

**Authors:** Jesse B. Adams, Stephen M. Bollens, John G. Bishop

**Affiliations:** 1 School of the Environment, Washington State University, Vancouver, Washington, United States of America; 2 School of Biological Sciences, Washington State University, Vancouver, Washington, United States of America; Stockholm University, SWEDEN

## Abstract

Invasive planktonic crustaceans have become a prominent feature of aquatic communities worldwide, yet their effects on food webs are not well known. The Asian calanoid copepod, *Pseudodiaptomus forbesi*, introduced to the Columbia River Estuary approximately 15 years ago, now dominates the late-summer zooplankton community, but its use by native aquatic predators is unknown. We investigated whether three species of planktivorous fishes (chinook salmon, three-spined stickleback, and northern pikeminnow) and one species of mysid exhibited higher feeding rates on native copepods and cladocerans relative to *P*. *forbesi* by conducting `single-prey’ feeding experiments and, additionally, examined selectivity for prey types with `two-prey’ feeding experiments. In single-prey experiments individual predator species showed no difference in feeding rates on native cyclopoid copepods (*Cyclopidae* spp.) relative to invasive *P*. *forbesi*, though wild-collected predators exhibited higher feeding rates on cyclopoids when considered in aggregate. In two-prey experiments, chinook salmon and northern pikeminnow both strongly selected native cladocerans (*Daphnia retrocurva*) over *P*. *forbesi*, and moreover, northern pikeminnow selected native *Cyclopidae* spp. over *P*. *forbesi*. On the other hand, in two-prey experiments, chinook salmon, three-spined stickleback and mysids were non- selective with respect to feeding on native cyclopoid copepods versus *P*. *forbesi*. Our results indicate that all four native predators in the Columbia River Estuary can consume the invasive copepod, *P*. *forbesi*, but that some predators select for native zooplankton over *P*. *forbesi*, most likely due to one (or both) of two possible underlying casual mechanisms: 1) differential taxon-specific prey motility and escape responses (calanoids > cyclopoids > daphnids) or 2) the invasive status of the zooplankton prey resulting in naivety, and thus lower feeding rates, of native predators feeding on invasive prey.

## Introduction

Worldwide, human impacts on freshwater and estuarine ecosystems facilitate the introduction and establishment of aquatic invasive species (AIS), resulting in significant economic and ecological impacts [1,2]. For example, in the Great Lakes (U.S.), loss of ecosystem services due to ship-borne AIS invasions was recently estimated at between $138 million and $800 million [3]. Aquatic species invasions generally occur as a result of human activities, including habitat alteration, aquaculture, exotic species trade or ship ballast-water releases, and they are a leading driver of global biodiversity loss [4–6]. Successful establishment of invaders largely depends on the suitability of invaded habitat, native community resistance and the propagule pressure of the invading organisms [7]. AIS are particularly problematic because detection, assessment and control are more costly and difficult in aquatic systems [8,9], leading to inadequate prevention and an incomplete understanding of invader impacts. Therefore, investigations of the interactions between native biota and AIS are crucial to gain better understanding of AIS impacts and to inform effective management strategies in the future.

The potential for AIS to alter food-webs is a major concern in many invaded freshwater and estuarine habitats [10–12]. Many studies have examined the effects of non-native predators on native prey populations, and invasive prey populations can have a variety of effects on native predators and food webs [10,13,14], but far fewer studies have examined the impacts of AIS as an alternative prey resource for native predators [13,15]. In particular, limited information exists on the suitability of invasive zooplankton prey as food resources for native predators [15–17], or of the ability of native aquatic predators to control invasive zooplankton populations [18,19]. However, changes in prey availability and predator consumption habits can have major impacts on native predator populations. For example, in the Great Lakes (US), alewife (*Alosa pseudoharengus*) and lake whitefish (*Coregonus clupeaformis*) populations experienced declines in consumption and body condition as their preferred prey, the native amphipod *Diporeia*, all but disappeared as system productivity (i.e., food resources) declined following invasion of the zebra mussel, *Dreissena polymorpha* [20–22]. In addition, some research predicts an additive effect of climate change and aquatic species invasions that will strengthen competitive and predatory interactions between AIS and native biota, leading to greater ecological change in the future [23,24]. Thus examining the effects of AIS on predator-prey interactions will improve our understanding of how trophic interactions affect the success of AIS, as well as how AIS may be changing aquatic communities and ecosystems.

Invasive zooplankton—released to estuaries and freshwaters via domestic and international shipping and boating—are a common occurrence worldwide [25], and in the U.S. Pacific Northwest (PNW) specifically [26]. The Columbia River drains 668,000 km^2^ of western North America, creating a freshwater-dominated estuary and discharging more water to the Pacific Ocean than any other river in North America [27]. In addition to supporting abundant wildlife and important salmon stocks, the Columbia River hosts major ports and hydroelectric production, with dams and impoundments that facilitate shipping (and thus colonization by AIS) far upriver. Several Asian copepods are currently established in the Columbia River [9,28,29]. Copepods are small planktonic crustaceans with diverse global distributions and diets, ranging from phytoplankton and bacteria to other zooplankton [30]. Along with other common zooplankton like rotifers and cladocerans, copepods are important consumers, as well as prey for fish and invertebrate predators, in freshwater and estuarine food-webs

A recent invader to western North America estuaries and rivers, the Asian calanoid copepod, *Pseudodiaptomus forbesi*–discovered in the San Francisco estuary in 1987 [31] and subsequently in the Columbia River estuary (CRE) in 2002 [32]—is now the dominant component of the CRE zooplankton community in late summer and early fall [28,29,33,34]. *P*. *forbesi* co-occurs with several native zooplankton species in the CRE, and seems to have replaced a closely related invasive calanoid copepod, *Pseudodiaptomus inopinus*. *P*. *forbesi* has also spread upstream into several reservoirs in the lower and middle parts of the Columbia River, where it can reach very high densities [35]; however, its interactions with higher trophic level consumers in the Columbia River system such as fish and macroinvertebrates is unknown.

Estuarine and freshwater predators such as fish and mysids are known to feed selectively on zooplankton prey [36–40]. However, we know of only two prior experimental studies of selective predation on both native and invasive zooplankton. A study by Meng and Orsi [41] examined selective predation by larval striped bass, *Morone saxatilis*, on native and invasive copepods in the San Francisco estuary and found that native copepods were captured more frequently than invasive copepods. Conversely, Bollens et al. [9] undertook selective predation experiments with a wide range of estuarine predators feeding on native and invasive copepods in the Chehalis River estuary, and found mostly neutral prey selectivity. Given these limited and variable results, there is a need for further studies of predator-prey dynamics of invasive zooplankton in large, human-impacted estuarine ecosystems (such as the CRE) to better predict and manage the impacts of these invasions.

We experimentally examined prey selection and prey-specific feeding rates of four native CRE predators—three species of planktivorous fishes and one species of mysid—on common CRE zooplankton prey in the laboratory using native cyclopoid copepods and cladocerans and the invasive calanoid copepod, *P*. *forbesi*. Unfortunately, controlling for taxonomic differences in native and invasive prey in the CRE was not possible because native calanoid copepods are not sufficiently abundant in the system to test directly against *P*. *forbesi*. Nevertheless, we were able to address our overarching objective—to investigate potential differences in feeding rates of common CRE predators between common native zooplankton and invasive *P*. *forbesi*. More specifically, we tested the hypothesis that native CRE predators feed on common native zooplankton prey at higher rates than on the invasive *P*. *forbesi*, reasoning that native predators might be naïve to the unique escape response of *P*. *forbesi* (i.e., occasional, high-speed swimming bursts or ‘jumps’ of calanoid copepods compared to the slower, more continuous motions of native zooplankton such as daphnids and cyclopoid copepods) or they might not visually recognize the invader as a potential food source as readily as native prey [41]. An alternative hypothesis was that *P*. *forbesi* might be naïve to native predators, and thus, more easily captured and consumed, in what Wanger et al. [18] termed the invasive-naïvety effect. Finally, we recognized a second alternative hypothesis, namely, that native predators might demonstrate no difference in feeding rates or selectivity for native zooplankton versus *P*. *forbesi*, which might indicate that some invasive zooplankton are a dietary substitute for native zooplankton prey in altered ecological systems (though with no presumptions of actual dietary suitability of the invader).

## Materials and Methods

### Ethics Statement

Our study strictly followed Institutional Animal Care and Use Committee (IACUC) of Washington State University (WSU) guidelines throughout and was approved by the WSU IACUC (permit number: 04288–001). All efforts were made to keep animals in good health and minimize stress and suffering. In the event of incurable illness, humane euthanasia with MS-222 using approved IACUC protocols for fish was utilized. Field collection permits were granted through National Marine Fisheries Service (permit number: 01-12-NWFSC81), Oregon Department of Fish and Wildlife (permit number: 17021), and Washington Department of Fish and Wildlife (permit number: 10–433) and fish were transported under permit by Washington Department of Fish and Wildlife (permit number: 5956-07-12).

### Study Design

Laboratory feeding experiments were used to test various CRE predators’ feeding rates and selectivity for different zooplankton prey taxa following protocols modified from Bollens et al. [9], Murtaugh [36], Bollens et al. [38], Avent et al. [39], and Bollens and Frost [42]. We utilized two different types of feeding experiments—one in which a single predator was presented with a fixed total number (i.e. no replacement) of a single prey-type at a time, and another in which a single predator was presented with two prey-types simultaneously. Both single and two-prey experiments were intended to simulate lower Columbia River conditions where one or two zooplankton species dominate the mesozooplankton community [29,34,35]. While both tests compare predation rates on alternative prey types, the single-prey experiments differed from the two-prey experiments by examining feeding rates in the absence of both direct and indirect interspecific prey interactions [43], while two-prey experiments explicitly test for selectivity among prey types. Comparing feeding rates in both single-prey and two-prey experiments may also permit inference of prey selection [44].

### Species Descriptions, Collection and Acclimation

Predation experiments were conducted 05-Sept-2012–28-Sept-2012 and 05-Aug-2013–30-Sept-2013. Experimental predators included adult three-spined stickleback (*Gasterosteus aculeatus*), juvenile northern pikeminnow (*Ptychocheilus oregonensis*) and adult mysid shrimp (*Neomysis mercedis*) collected from the CRE. Additionally, hatchery reared juvenile chinook salmon (*Oncorhynchus tshawytscha*) were obtained from the United States Geological Survey Western Fisheries Research Center near Cook, Washington. We note that hatchery-reared salmon do not experience live prey before being released to the wild, and we discuss this topic and its possible implications for our results below. All four of these predator species are planktivores that include copepods and/or cladocerans in their diet (*G*. *aculeatus* [45]; juvenile *P*. *oregonensis* [46]; juvenile *O*. *tshawytscha* [40,47]; and *N*. *mercedis* [9,48]). Furthermore, we chose these predator species because they all are common in the CRE during summer and spatially and temporally co-occur with each other and the zooplankton species (see below) used in this study [29,49–51]. The four predators used exhibit various types of species-specific feeding behaviors. For example, juvenile chinook salmon and northern pikeminnow cover large search areas by actively swimming in zigzag patterns while seeking prey [52]. On the other hand, three-spined sticklebacks tend to swim at slower speeds and often remain still, allowing prey to inadvertently approach before quickly attacking unsuspecting individuals at close distances [53]. *Neomysis mercedis* tends to associate with the benthos during daylight hours and swims actively into the pelagic zone to hunt in the evening by actively searching and chasing prey and then striking quickly once prey are near [54].

Wild fish were caught with a seine net (4.5 m long X 1.2 m high; 3.2 mm mesh size) and *N*. *mercedis* with a benthic sled (1.5 m hoop, 500 μm cod end). *N*. *mercedis* and three-spined stickleback*s* were obtained from tidally-influenced freshwater regions (Practical Salinity, *S* = 0.0) of the CRE and northern pikeminnow from the nearby lower Chehalis River (*S* = 0.0). Wild and hatchery fish ranged from approximately 3 to 6 cm total length and mysid shrimp 1 to 2 cm total length (see below for details). Predators were transported within five hours to our laboratory on the Washington State University Vancouver campus in coolers or buckets with aerated water and covered to minimize stress.

Fish were separated into groups by species and acclimated in the laboratory for a minimum of one week before use in experiments. Groups of fish were held in 38- or 56 -L aquaria filled with filtered estuary water (*S* = 0.0) and the water temperature was adjusted over 24 hrs. from the temperature at collection (19°±2°C) to a temperature of 17°C±1°C. Holding tanks contained gravel substrate, plastic plants for habitat and continuous aeration and filtration. Indirect overhead light was provided by 75W incandescent bulbs set on a timer to approximate the natural light cycle of the CRE in summer (14 h light and 10 h dark). During holding and acclimation, fish were fed a maintenance diet of approximately 4% total fish biomass per day, with wild fish receiving a mix of frozen Chironomidae larvae and *Artemia sp*., and salmon receiving hatchery feed (Rangen Salmon Grower aquaculture crumbles #1). The mysid predators, *N*. *mercedis*, were held in a 38-L aerated aquarium established with CRE water (*S* = 0.0) and benthic substrate and fed a mix of frozen Chironomidae larvae and *Artemia* sp. twice a week. Additionally, all predators were fed live mixed zooplankton once a week to ensure their ability to recognize live prey in experiments, but this happened no less than 48h before experiments to avoid predators recalling recent prior experiences with prey. Furthermore, we reasoned that this supplemental feeding was infrequent enough to avoid predators becoming familiar with any specific type of live prey. Predators were acclimated to these lab conditions for at least one week before experiments and only those predators that appeared healthy were used in experiments.

Experimental zooplankton prey species were the native cladoceran *Daphnia retrocurva*, two native cyclopoid copepods, *Diacyclops thomasi* and *Acanthocyclops* sp. (hereby referred to collectively as *Cyclopidae* spp.) and the invasive copepod *Pseudodiaptomus forbesi*. Our focus on the most abundant mesozooplankton species in the CRE allowed us to investigate rates of predation on a well-established invasive copepod relative to common native zooplankton prey in the CRE, but because there are no native Pseudodiaptomids, discerning differences in predation due purely to prey species origin (i.e. native versus invasive) versus purely taxonomic differences was beyond the scope of this study. Zooplankton were collected with a plankton net (0.3 m diameter mouth with 100 μm cod end; Sea-Gear model 9000) from 2–6 m depth from a dock in the upper CRE at Vancouver, Washington, and transported in 20-L buckets of estuary water (*S* = 0.0) back to the laboratory. Zooplankton were held for no more than 3 days in aerated 20-L buckets of unfiltered estuary water, and adjusted over 24 hrs. from the temperature at collection (19°±2°C) to 17°C ±1°C, with ambient light from a nearby window and overhead grow lamps (to maintain phytoplankton) on a 12-hour on, 12-hour off diel cycle.

### Feeding Experiments

Live zooplankton was sorted for use in experiments by pipette under a dissecting microscope with animals identified to the lowest practical taxonomic level. Most prey types were easily identifiable (e.g., head morphology of *Daphnia* spp.; antennae and carapace length of *P*. *forbesi*), however, the main morphological diagnostic distinguishing the two *Cyclopidae* species, *D*. *thomasi* and *Acanthocyclops* sp., is the relative position of a small spine located on the caudal rami. There is considerable intraspecific variation in the location of this spine, and it is extremely difficult to detect in live, free-swimming specimens, making assessment of moving copepods difficult without causing stress or injury to the animal. Furthermore, both cyclopoid species are of similar size and color and exhibit similar motility patterns, thus, we decided to combine them into a single prey category. For all zooplankton specimens, we used only non-ovigerous adult females, approximately 1.4–1.6 mm in length, and of similar pigmentation, to try to control for possible predator selection based on size or pigmentation [42,55,56]. We analyzed 500 preserved specimens of each prey type (*D*. *retrocurva*, *Cyclopidae* spp. and *P*. *forbesi*) remaining from concluded experiments in order to verify the accuracy of prey identification, size (estimated to ±0.1mm using an ocular micrometer) and the proportions of the two species combined into the *Cyclopidae* spp. prey category (to assess the potential for bias in results due to size-selective feeding and intra-genus prey differences, respectively).

In each experiment, individual predators were allowed to feed for a set period of time—calibrated to remove 50–75% of prey—whereupon predators were then removed and the remaining zooplankton counted and identified. Ending the trial with 25–50% of prey remaining ensured that prey density remained in an intermediate range throughout the feeding trial, thereby approximating natural conditions in a small-scale homogenous prey environment [57] and avoiding the potential for non-selectivity at low prey densities [55], while also avoiding total prey depletion or satiation [53,58]. We estimated feeding rates in preliminary experiments for each predator species, which we then used in all subsequent experiments. This was done by carefully observing the prey strike frequency for each predator in three preliminary five-minute experiments using a mix (100 total) of the three prey types (i.e., *D*. *retrocurva*, *Cyclopidae* spp. and *P*. *forbesi*). We then extrapolated to obtain the following approximate times for each predator type to consume a maximum of 50–75% of the total prey items: three-spined stickleback, 45 minutes; northern pikeminnow, 30 minutes; chinook salmon, 30 minutes.

Fish predators were starved for ~24 hrs. before experiments to ensure adequate hunger and acclimated for ~2 hrs., individually, in a 38-L glass experimental aquarium open on the top, wrapped on all sides with black plastic and filled with 30 L of 17°C ±1°C, filtered and aerated estuary water (*S* = 0.0). No aeration was provided during experiments to avoid the effects of turbulent mixing on prey distributions within the arena [59]. Moreover, to simulate a pelagic environment, no refuge or substrate was used. All fish feeding experiments were conducted during daylight hours, with identical lighting to the holding tanks described above, and each predator individual was used in only a single experiment. Next, either 100 individuals of a single prey-type or 50 individuals each of two prey-types (depending on whether it was a single-prey or two-prey experiment, respectively) were introduced to the predator by slowly pouring the prey from a beaker into the center of the tank. After being allowed to feed unobserved for the predetermined amount of time, the predator was then removed and rinsed off into the tank, and the entire tank contents were then drained and rinsed through a 35-μm sieve. Remaining zooplankton was then carefully rinsed from the sieve into a jar, preserved in 10% formalin, and the remaining number of each prey type determined using a dissecting microscope. Missing zooplankton was presumed eaten. For single-prey experiments, five replicated feeding trials were performed for each of three fish predators (northern pikeminnow, chinook salmon and three-spined stickleback) on each of two prey taxa (*P*. *forbesi* and *Cyclopidae* spp.) for a total of 30 fish predator, single-prey experiments. For two-prey experiments, ten replicate experiments were done for each of two fish predators (northern pikeminnow and chinook salmon) presented in one set of experiments with native *Cyclopidae* spp. and invasive *P*. *forbesi*, and in another set of experiments with native *D*. *retrocurva* and invasive *P*. *forbesi*. Additionally, seven replicated experiments were conducted with three-spined stickleback paired with native *Cyclopidae* spp. and invasive *P*. *forbesi*, for a total of 47 fish predator, two-prey experiments. This yielded a total of 77 fish predator-zooplankton prey experiments; however, it was not possible to run experiments on all predator-prey combinations due to the limited time during which *P*. *forbesi* was available and the constraints of time and resources needed to run multiple, concurrent experiments.

Individual mysid predators (*N*. *mercedis*) were starved and acclimated (simultaneously) as were fish predators, but in 4-L Erlenmeyer flasks filled with filtered and aerated estuary water (*S* = 0.0) held in a 17°C ±1°C bath wrapped on all sides with black plastic and open on top. As in the fish experiments, no aeration, refuge or substrate were used. Indirect overhead 75W incandescent bulbs on a timer simulated the natural diel light cycle of the CRE in late summer (14 h light and 10 h dark). Around mid-day, either 50 of one prey-type or 25 each of two different prey-types (for a total of 50 prey items) were introduced to a mysid by carefully pouring the prey from a beaker into the flask. The mysid was then allowed to feed unobserved for a period of 24 hrs., after which all contents of the flask were treated as in the fish experiments. In mysid single-prey experiments, five replicates were done for each of two prey types (native *Cyclopidae* spp. and invasive *P*. *forbesi*), and in the mysid two-prey experiments (using native *Cyclopidae* spp. and invasive *P*. *forbesi*), seven replicated experiments were conducted, for a total of 17 mysid predator-zooplankton prey experiments.

### Statistical Analyses

For single-prey data, we conducted two sample t-tests to test the null hypothesis of no difference in mean feeding rate of native versus non-native copepods by each predator type. Here we define rate as the number of prey items consumed during the experiment, which was of a fixed duration for each predator type. Because of the modest number of replicates for each predator species, we also evaluated feeding rates considering all four predators simultaneously using a linear model (lm() in the R computing environment), with feeding rate as the dependent variable, and prey type and predator species as the explanatory variables. Interaction terms between prey and predator species did not improve the model AIC and were discarded. Because chinook salmon were hatchery raised, and not fed live prey prior to our acclimation period, while the other three predators were wild-caught, and notable behavioral differences between wild and hatchery-reared salmon have been observed [60,61], we considered a second model that included only the three wild-caught predator species. The choice to separately consider the three wild caught species was reinforced by our observation of notably high variation among hatchery-reared chinook salmon individuals in total prey items consumed in the two prey experiments (coefficients of variation for total prey eaten/predator individual: Neomysis: 25.3, Chinook: 59.0, Pikeminnow: 36.0, Stickleback: 35.9), although CVs were similar among predators in one-prey experiments. For the combined analysis, feeding rates were standardized across predators by dividing by the number of prey items available. Residual variation was Gaussian distributed (assessed visually using a qqplot), and residuals showed no relationship to fitted values.

For two-prey experiments we tested the null hypothesis of no difference in average predation rates on paired prey items using paired t-tests. We also analyzed selection among prey types by calculating a selectivity index for *P*. *forbesi* for each individual predator using equation 18 of Manly (1974):

β=ln( rFAF)ln( rFAF)+ln( rNAN)

where r_F_ denotes the number of *P*. *forbesi* remaining uneaten at the conclusion of the experiment, A_F_ is the number of *P*. *forbesi* available, and r_N_ and A_N_ are the corresponding values for the native prey item, either *D*. *retrocurva* or *Cyclopidae*. Analysis of β is particularly recommended for experiments where prey are consumed without replacement [62–64]. Values of β< 0.5 indicate selection against *P*. *forbesi*, while β = 0.5 indicates neutral selectivity. For each experiment, we used a one-sample t-test to test for departure from neutral selectivity [65,66]. We also conducted chi-square analyses of 2x2 contingency tables for two prey experiments, another commonly used approach for analyzing prey consumption data, however this additional analysis yielded no major differences with the other analyses in the overall results (S1 Table).

All single-prey and two-prey data sets were assessed for normality using Shapiro-Wilk normality tests and for homoscedasticity of variances using an F test. Assumptions were met except in one case with a significant Shapiro-Wilk test, where an additional non-parametric Wilcoxon signed-rank test was conducted to test for equality of means. Because predator size often affects a predator’s feeding selectivity through physical constraints such as gape limitations and swimming speed, we were careful to use predators of similar size within each species. Nevertheless, as some size variation was unavoidable, we investigated whether differences in individual predator lengths might account for differences in consumption of different prey species using linear regressions of prey consumption and β as a function of predator length (in both single- and two-prey experiments). All statistical analyses were performed in R 3.0.2.

## Results

### Prey Identification and Size

From a sample of 500 remaining copepods, the *Cyclopidae* spp. consisted of 72.4% *Acanthocyclops* sp. and 27.6% *Diacyclops thomasi*, and were 1.4–1.6 mm non-ovigerous females of nearly the same color (translucent yellow-brown) (S2 Table). Although possibly influenced by selective predator feeding during the experiments, these results suggest that our efforts to restrict variation in cyclopid prey size and color were successful. Additionally, *post hoc* analyses of other prey concluded accurate identification of species, gender and non-ovigerity as well as similar intraspecific size and coloration (S2 Table).

### Single-Prey Experiments

**Fig 1:**
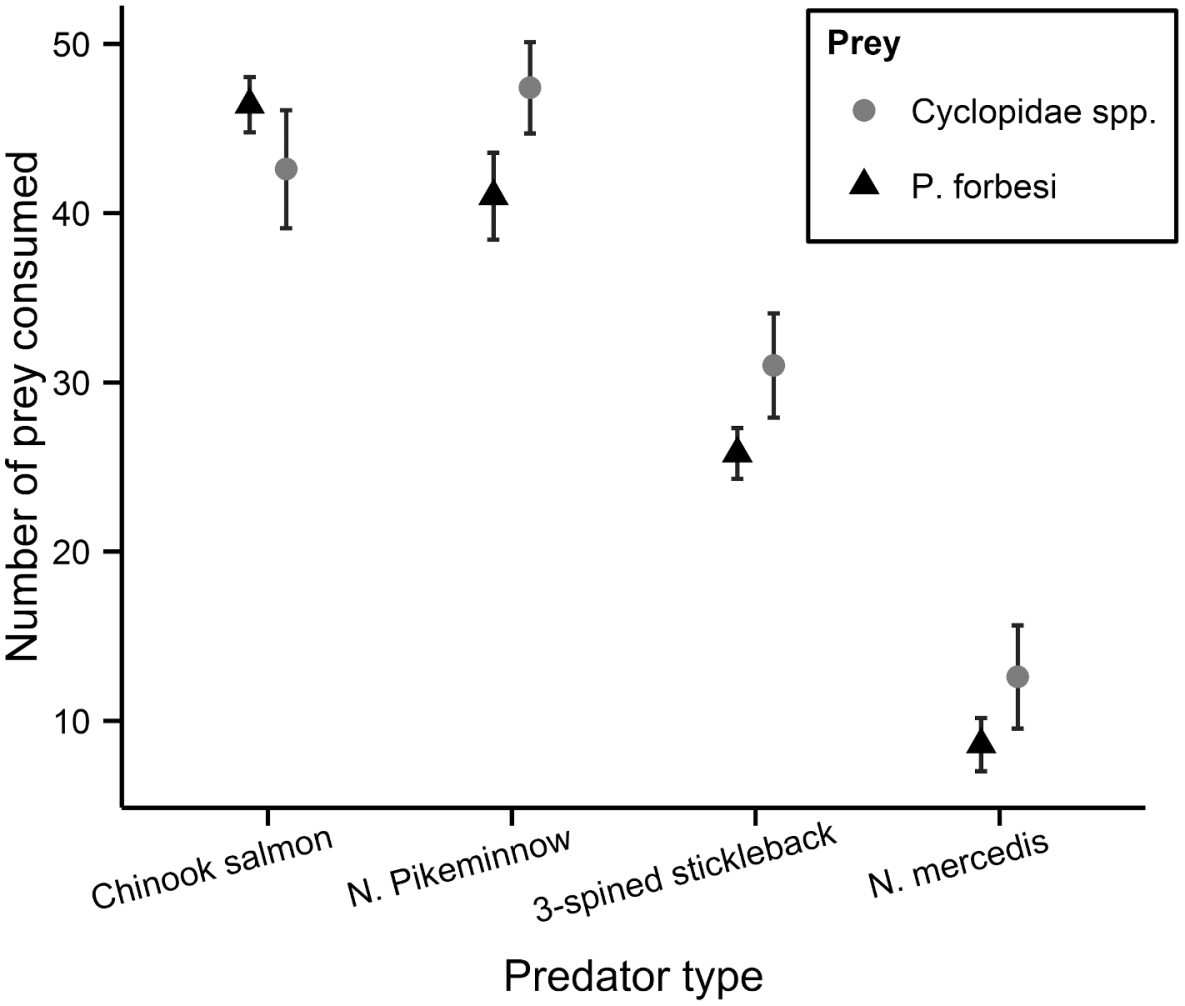
Mean and standard error of *Pseudodiaptomus forbesi* and native copepod prey consumed by four different predator types in single-prey experiments. Black triangles represent the invasive copepod, *Pseudodiaptomus forbesi*, and gray circles represent native copepods, *Cyclopidae* spp.

Two sample *t*- tests indicated no differences in the rates of consumption of native copepods vs. the invasive *P*. *forbesi*, suggesting that the predators did not consume prey types at different rates when offered only one kind of prey at a time (Fig 1; S3 and S4 Tables). However, the number of trials for each predator was modest (*n* = 5 per prey type) and all three wild-caught predator species trended toward higher feeding rates on native cyclopoid copepods (Fig 1 and S4 Table). When all four predators were analyzed in one model, there was still no evidence of feeding rate differences, nor any difference among predators (i.e. predator x prey interaction effects were non-significant). However when Chinook salmon were omitted and only the three wild-caught predators were considered, feeding rates were 7% higher on native cyclopoid copepods than on invasive *P*. *forbesi* (Effect of prey type: F_df = 1,26_ = 5.7, *P* = 0.02). While pikeminnows exhibited a higher overall feeding rate than other predators (F_df = 2,26_ = 25, *P* <0.0001), the lack of significant predator x prey interaction effects indicates similar decreases in feeding rates by all three wild predators on the invasive *P*. *forbesi*. In addition, there were no significant effects of predator length on total prey consumed or on prey-specific feeding rates, as expected given the relatively small differences in sizes between individual predators within a species (means + S.E.: chinook: 46.8 + 1.2mm; pikeminnow: 56.1 + 0.9 mm; stickleback: 40.8 + 0.9 mm; Neomysis: 14 + 0.5 mm) and our modest sample sizes.

### Two-prey experiments

**Fig 2:**
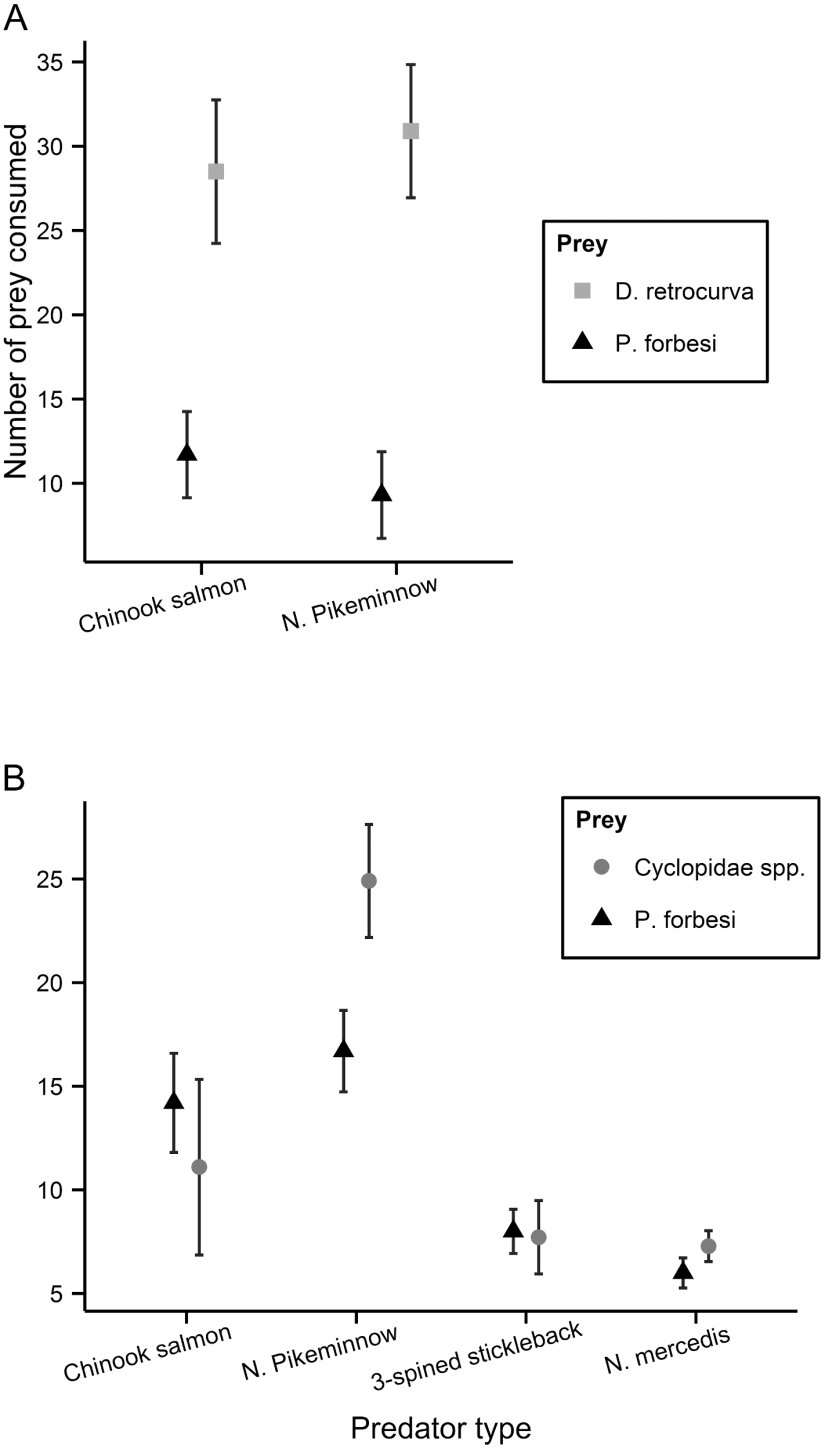
Mean and standard error of *Pseudodiaptomus forbesi* and native prey consumed by four different predator types in two-prey experiments. (A) Black triangles represent the invasive copepod, *Pseudodiaptomus forbesi*, and gray squares represent native cladocerans, *Daphnia retrocurva*. (B) Black triangles represent the invasive copepod, *Pseudodiaptomus forbesi*, and gray circles represent native copepods, *Cyclopidae* spp.

Predators offered a choice between the native cladoceran *Daphnia retrocurva* and the non-native copepod *P*. *forbesi* overwhelmingly selected *D*. *retrocurva* in two-prey experiments; however, only chinook salmon and northern pikeminnow were tested. Chinook salmon had higher feeding rates on *D*. *retrocurva* relative to *P*. *forbesi* (t = 3.7, df = 9, P = 0.005) and showed strong positive selection for *D*. *retrocurva* (Fig 2A; β = 0.28, t = 11.8, df = 9, P<0.005; S3 and S5 Tables). Likewise, northern pikeminnow feeding rates were higher on *D*. *retrocurva* (t = 7.1, df = 9, P<0.0001), which translated into very strong positive selection for *D*. *retrocurva* versus *P*. *forbesi* (β = 0.14, t = 4.1, df = 9, P<0.0001; Fig 2A, S3 and S5 Tables). In addition, northern pikeminnow exhibited significantly higher feeding rates on the native copepods over *P*. *forbesi* (t = 2.4, df = 9, P = 0.04), resulting in positive selection for native copepods over *P*. *forbesi* (β = 0.39), though the departure from neutrality was only marginally significant (t = 2.0, df = 9, P = 0.08; Fig 2B, S3 and S5 Tables). Chinook salmon was the only predator to show some tendency for selection of *P*. *forbesi* over the native cyclopoid copepods, but because of high variance in β among fish, this trend was not significant (β = 0.67, t = 1.8, df = 9, P = 0.10). There was no evidence for differences in feeding rate or selection between native cyclopoid copepods and *P*. *forbesi* for the other predators tested individually (Neomysis β = 0.44, p = 0.14; sticklebackβ = 0.46, p = 0.6), nor in the model including all 4 predators or the 3 wild-caught predators. Finally, there was no relationship between predator length and the selectivity index β (linear regression, *P* > 0.20 for all cases), again reflecting low within-species variation in length.

## Discussion

Our results indicate that diverse taxa of native predators in the CRE are capable of feeding on the invasive copepod, *P*. *forbesi*, although some (but not all) of these predators feed on native zooplankton at higher rates. However, the underlying reason for these instances of higher predation on native over invasive zooplankton prey is not clear, and could be due to one or both of two different mechanisms: differential taxon-specific prey motility and escape responses, or the invasive status of the zooplankton prey and thus the naivety of native predator interactions with invasive prey.

Prey selection is well documented in predatory animals and is often an important factor in structuring prey populations and food webs [67–71]. A common factor in such studies is selection of prey by size according to optimal foraging strategies [55,72], so we sought to minimize size-selection as a factor by using similar-sized zooplankton across taxa. However, predator-prey interactions may be mediated by a number of factors that are difficult to disentangle. While the taxonomic groups of native zooplankton and invasive *P*. *forbesi* in our experiments differed, we chose functionally similar taxonomic groups that occur in appreciable numbers in the CRE. Since native Pseudodiaptomids do not inhabit freshwater areas of the CRE, we felt that using abundant native zooplankton allowed us to best investigate potential invasion impacts on native CRE predators. Although both terrestrial and aquatic field studies provide evidence that native predators may feed at different rates on or select between native and invasive prey [13,15,73], few experimental studies have tested this [9,14,65,74]. While the precise reasons for differences in feeding rates or selection of specific prey types may be unknown—as it was in our study—whether or not predators select for or feed at higher rates on one type of prey (i.e. native or invasive) over another, or adapt in any way to the presence of invasive prey (e.g. [75,76]) is important to understanding the impacts of zooplankton invasions on native predator populations, and ultimately the invaders’ effects on native food-webs.

We found that some native aquatic predators capture native zooplankton at higher rates than they do the invasive *P*. *forbesi* when both prey are offered to a predator together; for example, in two-prey experiments juvenile northern pikeminnow selected native copepods (*Cyclopidae* spp.) over invasive copepods (*P*. *forbesi*). Predation rates on *Cyclopidae* spp. were also higher in single prey experiments for the 3 wild-caught predators (Fig 1), with good statistical support when they were considered simultaneously. Additionally, northern pikeminnow strongly selected native cladocerans, *D*. *retrocurva*, over the invasive copepod, *P*. *forbesi*; a finding consistent with other observations showing that planktivorous fish consume *Daphnia* spp. at higher rates than similar-sized calanoid copepods [77]. Several previous studies have examined the feeding habits of adult northern pikeminnow, particularly with regard to their voracious appetite for juvenile salmon [51,78–80], but there is very little information on this species’ diet early in its life history beyond general consumption of micro-crustaceans [46]. Our finding of selective feeding favoring native zooplankton over the invasive *P*. *forbesi* indicate that early life history stages of northern pikeminnow may be negatively affected by displacement of native prey by *P*. *forbesi* in the CRE.

One potential explanation for northern pikeminnow selecting against *P*. *forbesi* could be that this predator is poorly adapted to catching the invasive copepod. In other predator-prey systems, researchers have found evidence of ‘adaptive lag-time’ in native predators’ abilities to effectively acquire novel invasive prey [13]. For example, native whelks feeding on two different rocky intertidal mussels were less adapted (thus, less successful) at feeding on the more recent invasive mussel compared to the long established mussel species [81]. Indeed, zooplankton evasion can play a significant role in the outcome of predator-prey interactions [11,82,83]. Similar to Meng and Orsi [41], we qualitatively observed species-specific predator evasion and avoidance behaviors, as cylopoid copepods seemed more conspicuous and predictable in their behavior, constantly moving in short, erratic pulses, whereas the calanoid copepod, *P*. *forbesi*, seemed more inconspicuous and unpredictable, mostly gliding with minimal motion, but punctuated by periodic high-speed swimming bursts in different directions. The cladoceran, *D*. *retrocurva*, seemed to have by far the least effective predator evasion behavior, as it was much slower and less agile than either copepod species, and may have also been more conspicuous than copepods due to its rounder shape [83]. Nevertheless, we recognize that the visibility and susceptibility of these prey items may vary somewhat in our experimental tanks (using standing water) vs nature (e.g., higher flow and turbulence, and thus potentially turbidity, in the CRE).

Juvenile chinook salmon, much like northern pikeminnow, also strongly selected for *D*. *retrocurva* over the invasive *P*. *forbesi*. This increased feeding on cladocerans over copepods was consistent with previous zooplankton prey selection studies [83] and diet studies of juvenile salmon in the CRE [47]. However, we found no significant differences in feeding rates or selection by chinook salmon with respect to native cyclopoid copepods versus the invasive calanoid copepod *P*. *forbesi*.

Despite our findings of neutral selectivity between *P*. *forbesi* and native copepods by juvenile chinook salmon, there is little field evidence that invasive calanoid copepods occur in their diet [48]. There are several reasons this might be the case. First, smaller juvenile predators may be underrepresented in field sampling, as sampling for diet analyses becomes more difficult to conduct on smaller fishes [84], potentially biasing against planktivorous stages of some juvenile fishes. Second, previous field sampling may not have coincided with the seasonal and spatial overlap of predators and invasive zooplankton populations. For example, fish may shift from littoral zone resources to pelagic resources, such as plankton, as the availability of aquatic insect larvae and other benthic invertebrates becomes more limited throughout summer and into fall [50,85].

Interpreting the results of our experiments on juvenile chinook salmon warrants caution for two other reasons as well. First, hatchery-reared salmon are known to exhibit abnormal feeding (and other) behaviors [60,61] that may have contributed to the outcome of our feeding experiments with chinook salmon. Second, it is possible that our experimental design utilizing only a single predator in the arena may have influenced chinook salmon feeding behavior, as they are commonly known to aggregate or school in estuaries and streams [52,86]. Ultimately, further field and experimental studies of wild juvenile salmon diets are needed to determine if our results with hatchery-reared juveniles are applicable to wild salmon.

Differences in northern pikeminnow and chinook salmon selectivity for native *Cyclopidae* spp. and the invasive copepod *P*. *forbesi* (i.e., positive vs. neutral selectivity for *Cyclopidae* spp., respectively) is interesting in the context of the life histories and predator-prey dynamics of these two fishes. Smaller fishes and subyearling fish are at great risk of predation by larger piscivorous fishes [87,88]. This is particularly a concern in the Pacific Northwest of the U.S., where predation on juvenile chinook salmon by adult northern pikeminnow is common [79] and size of northern pikeminnow strongly determines when the switch from invertebrate to fish prey occurs [51,80]. Abundant and easily utilized prey resources may directly enhance juvenile fish growth, thereby potentially decreasing their risks of predation by larger fish [46,79]. Alternatively, indirect benefits to juvenile fish may arise via stunted growth of potential predators from a change in dominance from desirable prey (e.g. *Daphnia* and cyclopoid copepods) to undesirable prey (e.g. calanoid copepods) as mediated by dietary overlap and food limitation [14,15]. Such variable success in the selection of different prey types by different predators might contribute to differential growth rates [89], which may in turn have broader effects on food webs [51].

The third fish predator that we examined, three-spined stickleback, showed a higher (but non-significant) feeding rate on native copepods relative to *P*. *forbesi* in one-prey experiments, which contributed to the significantly higher rate of predation on native copepods across wild-caught predators (Fig 1); however, there was no evidence of prey selectivity of three-spined sticklebacks in two-prey experiments. Given differences in our results between stickleback (weak or neutral selectivity) and the northern pikeminnow (selective feeding on the native cyclopids), it is notable that sticklebacks utilized a very different type of search and attack behavior than the other two fishes. Whereas chinook salmon and northern pikeminnow actively swam around the tank searching for prey, sticklebacks used mostly a sit-and-wait approach, typically allowing prey to come within striking distance before attacking. Furthermore, active searching by chinook salmon and northern pikeminnow may have alerted prey to initiate an evasive response, resulting in predators capturing prey based, at least partly, on prey-specific evasion responses [83]. In comparison, three-spined stickleback behavior consisted of the fish `hovering’ near the bottom of the tank by rapidly beating its pectoral fins, and waiting until prey were near before thrusting with its caudal fin and quickly consuming prey. We hypothesize that this sit-and-wait predatory mode and swift strike of sticklebacks may have resulted in less time for prey to react compared to the ‘active search’ predatory mode used by the other two fish species. Therefore, even though native cyclopoid copepods and invasive *P*. *forbesi* in our experiments appeared to have somewhat different mobility patterns and evasive behaviors, this may not have had a great effect on three-spined sticklebacks’ capture efficiency [90] as their predator-prey interactions might have relied more on chance.

The mysid *N*. *mercedis* showed a non-significant trend toward a higher feeding rate on native copepods relative to *P*. *forbesi* in both the one- and two-prey experiments. These results, while consistent with other findings that copepods are often important prey in mysid diets [9,48], do not provide strong evidence that *N*. *mercedis* feeds selectively or at significantly different rates on different types of zooplankton, in contrast to some other studies [9,36]. Indeed, Bollens et al. [9] found *N*. *mercedis* selected native calanoid copepods (*Acartia [Acartiura]* sp.) over invasive calanoid copepods (*P*. *inopinus)*. However, in related field studies, *P*. *inopinus* was nevertheless found to comprise a large proportion of wild mysids’ diet [9,48]. To explain this discrepancy between their selection experiments and field observations, Bollens et al. [9] suggested that diel vertical migration (DVM) behavior, and the resulting spatial and temporal overlap between of *N*. *mercedis* and *P*. *inopinus*, may have resulted in different prey selection in laboratory experiments than in the wild, as vertical structure to allow for DVM was not incorporated into their laboratory experiments. DVM can be an important factor in affecting pelagic predator-prey interactions [91,92], and while beyond the scope of the current study, is something we recommend be incorporated into future studies.

In summary, we experimentally examined feeding rates of four native predators from the CRE on native zooplankton and the invasive copepod *P*. *forbesi*. Since we found no native calanoid copepods comparable to *P*. *forbesi* in any significant quantities in the CRE, we were unable to test differential feeding rates and selectivity of native vs. invasive zooplankton prey within a specific taxonomic group (e.g., calanoid copepods), and instead had to rely on testing for predator selection and feeding rates between slightly different zooplankton prey taxa. We found that some (but not all) native predators feed selectively on native zooplankton (*D*. *retrocurva* and *Cyclopidae* spp.) vs. the invasive calanoid copepod *P*. *forbesi*, which can most likely be attributed to one (or both) of two possible underlying casual mechanisms: 1) differential taxon-specific prey motility and escape responses (calanoids > cyclopoids > daphnids) or 2) the invasive status of the zooplankton prey resulting in naivety, and thus lower feeding rates, of native predators feeding on invasive prey. Additional prey-specific differences, such as palatability or energy content, might also be factors, but we are unaware of any such data for our study organisms, and these factors were beyond the scope of our study. In any event, we found that invasive calanoid copepods may provide a suitable alternative prey to native cyclopoid copepods for some aquatic predators. This last result is consistent with findings in the San Francisco estuary, where during certain times of the year the dominant dietary constituent of the threatened delta smelt (*Hypomesis transpacificus*) was the invasive copepod *P*. *forbesi* [93]. With the invasive *P*. *forbesi* the overwhelmingly dominant mesozooplanker during late summer and early fall in the CRE [29,33–35], the prolific copepod invader may also be impacting native predators there as well.

Furthermore, we found differences in selection patterns for different predator species, suggesting that reduced availability of native prey caused by zooplankton invasions may have species-specific effects on native predators. Several areas of additional research, such as the nutritional value of zooplankton prey, the functional responses of native predators, the degree of spatial overlap between predators and prey (e.g., as mediated by DVM), and the diet and selective feeding behaviors (if any) of zooplankton themselves could further elucidate the role of invasive zooplankton in native aquatic food webs. Such additional studies will be necessary to fully understand the impacts of invasive species, and aid in the management of native biota in heavily invaded ecosystems.

## Supporting Information

S1 Table Results of 2x2 contingency table χ^2^ analysis for two-prey experiments. (PDF)

S2 Table Mean (± SE) sizes of zooplankton prey used in experiments. (PDF)

S3 Table Results of two sample t-tests for single-prey experiments and paired t-tests and selection index for two-prey experiments. (PDF)

S4 Table Number of each prey type consumed in single-prey experiments. (PDF)

S5 Table Number of each prey type consumed in two-prey experiments. (PDF)
