# Supplementary material for: Predation on the Invasive Copepod, Pseudodiaptomus forbesi, and Native Zooplankton in the Lower Columbia River: An Experimental Approach to Quantify Differences in Prey-Specific Feeding Rates
Source: PLoS One. 2015 Nov 30;10(11):e0144095. doi: 10.1371/journal.pone.0144095 (PMC4664400; doi:10.1371/journal.pone.0144095)
Supplement: S1 Table — (PDF) [file pone.0144095.s001.pdf]

**S1 Table 1. Results of 2x2 contingency table $\chi^2$ analysis for two-prey experiments (DF=1)**

| **Predator** | **Prey** | $\chi^2$ | ***P*** |
|---|---|---|---|
| Chinook Salmon | Cyclopidae ~ P. forbesi | 3.8 | 0.051 |
| | *D. retrocurva* ~ P. forbesi | 70.2 | < 0.0001 |
| N. Pikeminnow | Cyclopidae ~ P. forbesi | 16.2 | < 0.0001 |
| | *D. retrocurva* ~ P. forbesi | 116.1 | < 0.0001 |
| Three-spined stickleback | Cyclopidae ~ P. forbesi | 0.04 | 0.85 |
| *N. mercedis* | Cyclopidae ~ P. forbesi | 0.87 | 0.35 |
