# Supplementary material for: Predation on the Invasive Copepod, Pseudodiaptomus forbesi, and Native Zooplankton in the Lower Columbia River: An Experimental Approach to Quantify Differences in Prey-Specific Feeding Rates
Source: PLoS One. 2015 Nov 30;10(11):e0144095. doi: 10.1371/journal.pone.0144095 (PMC4664400; doi:10.1371/journal.pone.0144095)
Supplement: S2 Table — (PDF) [file pone.0144095.s002.pdf]

S2 Table 2. Mean (± SE) sizes of zooplankton prey used in experiments. A total of 500 individuals of each prey type from the remaining uneaten prey were sampled across all experiments to estimate mean sizes of prey and the relative proportion of *Acanthocyclops* sp. and *Diacyclops thomasi* within the *Cyclopidae* spp. prey group.

**Prey**	***N***	**Mean size ± SE (mm)**
Daphnia retrocurva	500	1.4 ± 0.02
Cyclopidae spp.	500	1.5 ± 0.01
Acanthocyclops sp.	[362]	[1.5 ± 0.02]
Diacyclops thomasi	[138]	[1.5 ± 0.03]
Pseudodiaptomus forbesi	500	1.6 ± 0.01
