# Supplementary material for: Predation on the Invasive Copepod, Pseudodiaptomus forbesi, and Native Zooplankton in the Lower Columbia River: An Experimental Approach to Quantify Differences in Prey-Specific Feeding Rates
Source: PLoS One. 2015 Nov 30;10(11):e0144095. doi: 10.1371/journal.pone.0144095 (PMC4664400; doi:10.1371/journal.pone.0144095)
Supplement: S3 Table — (PDF) [file pone.0144095.s003.pdf]

S3 Table 3. Results of two sample t-tests for single-prey experiments and paired t-tests and selection index for two-prey experiments. DF=8 for single prey experiments. DF=9 for two-prey experiments except DF=6 for stickleback and *N. mercedis*.

		Single-Prey		Two-Prey, Feeding rates		Two prey, Selection index	
**Predator**	**Prey**	***t***	***P***	***t***	***P***	***t***	***P***
Chinook Salmon	Cyclopidae ~ P. forbesi	0.99	0.35	0.98	0.35	1.8	0.10
	D. retrocurva ~ P. forbesi	-	-	3.74	0.005	11.8	<0.005
N. Pikeminnow	Cyclopidae ~ P. forbesi	1.52	0.17	2.34	0.04	2.0	0.08
	D. retrocurva ~ P. forbesi	-	-	7.10	< 0.0001	4.1	<0.0001
Three-spined stickleback	Cyclopidae ~ P. forbesi	1.72	0.12	0.14	0.89	0.6	0.56
N. mercedis	Cyclopidae ~ P. forbesi	1.17	0.28	1.72	0.14	1.7	0.14
