# Supplementary material for: Predation on the Invasive Copepod, Pseudodiaptomus forbesi, and Native Zooplankton in the Lower Columbia River: An Experimental Approach to Quantify Differences in Prey-Specific Feeding Rates
Source: PLoS One. 2015 Nov 30;10(11):e0144095. doi: 10.1371/journal.pone.0144095 (PMC4664400; doi:10.1371/journal.pone.0144095)
Supplement: S4 Table — (PDF) [file pone.0144095.s004.pdf]

S4 Table 4. Number of each prey type consumed in single-prey experiments. *N* = 100 total prey items at start of experiments.

**Predator**		Chinook salmon		Northern pikeminnow		Three-spined stickleback		Neomysis mercedis	
**Prey**		Cyclopidae	P. forbesi	Cyclopidae	P. forbesi	Cyclopidae	P. forbesi	Cyclopidae	P. forbesi
**Replicate**	***1***	44	51	55	38	39	25	17	8
	2	32	44	43	44	22	23	9	4
	3	38	46	48	42	35	31	5	7
	4	47	49	51	33	33	27	22	13
	5	52	42	40	48	26	23	10	11
