# Supplementary material for: Predation on the Invasive Copepod, Pseudodiaptomus forbesi, and Native Zooplankton in the Lower Columbia River: An Experimental Approach to Quantify Differences in Prey-Specific Feeding Rates
Source: PLoS One. 2015 Nov 30;10(11):e0144095. doi: 10.1371/journal.pone.0144095 (PMC4664400; doi:10.1371/journal.pone.0144095)
Supplement: S5 Table — (PDF) [file pone.0144095.s005.pdf]

S5 Table 5. Number of each prey type consumed in two-prey experiments. *N* = 100 total prey items at start of experiments (50 of each prey type).

**Predator**		Chinook salmon		Northern Pikeminnow		Chinook salmon		Northern pikeminnow		Three-spined stickleback		Neomysis mercedis	
**Prey pair**		D. retrocurva	P. forbesi	D. retrocurva	P. forbesi	Cyclopidae	P. forbesi	Cyclopidae	P. forbesi	Cyclopidae	P. forbesi	Cyclopidae	P. forbesi
**Replicate**	***1***	13	2	26	3	1	12	31	23	12	7	8	6
	2	45	3	25	8	0	13	6	13	7	10	10	8
	3	39	25	24	3	34	19	22	29	2	6	7	9
	4	2	9	45	14	18	28	21	23	5	13	4	5
	5	23	10	44	25	1	7	26	11	14	9	7	4
	6	24	13	40	0	12	11	26	13	11	6	9	6
	7	37	7	27	13	4	14	35	15	3	5	6	4
	8	38	22	33	10	34	24	28	11	-	-	-	-
	9	38	6	41	17	0	11	19	17	-	-	-	-
	10	26	20	4	0	7	3	35	12	-	-	-	-
